# Obesity-induced elevated palmitic acid promotes inflammation and glucose metabolism disorders through GPRs/NF-κB/KLF7 pathway

**DOI:** 10.1038/s41387-022-00202-6

**Published:** 2022-04-20

**Authors:** Tongtong Qiu, Xin Yang, Jingzhou Wang, Chongge Pan, Xiaolong Chu, Jianyu Xiong, Jianxin Xie, Yongsheng Chang, Cuizhe Wang, Jun Zhang

**Affiliations:** 1 Medical College of Shihezi University, Bei-Er-Lu, Shihezi, 832000 Xinjiang China; 2 Department of Physiology and Pathophysiology, Tianjin Medical University, Tianjin, 300000 China

**Keywords:** Fat metabolism, Fatty acids

## Abstract

**Objective:**

Our previous results have shown that obesity-induced excessive palmitic acid (PA) can promote the expression of KLF7, which plays a vital role in regulation of inflammation, glucose metabolism. But the exact mechanism of PA up-regulating the expression of KLF7 is not clear yet. This study is intend to explore whether PA promoting KLF7 expression through GPRs/NF-κB signaling pathway, causing inflammation and glucose metabolism disorders.

**Methods:**

Cells were blocked GPRs/NF-κB under PA stimulation in vitro to demonstrate the molecular mechanism of PA up-regulates KLF7 expression. The regulatory effect of p65 on KLF7 was detected by luciferase reporter gene assay. Blocking GPRs/NF-κB in diet-induced obesity mice to detect the expression of KLF7, inflammatory cytokines and glucose metabolism related factors, clarifying the effects of GPRs/NF-κB on KLF7 in vivo.

**Results:**

In 3T3-L1 adipocytes and HepG2 cells, PA could up-regulate the expression of KLF7 by promoting the GPR40/120-NF-κB signaling pathway, leading to inflammation and reduced glucose consumption (*p* < 0.05 for both). Luciferase reporter gene assay and ChIP assay showed that p65 could transcriptionally up-regulates the expression of KLF7. In high-fat diet (HFD) mice, after intraperitoneal injection of GPR40 or GPR120 blocker, the levels of p-p65 and KLF7 in epididymal white adipose tissue and liver were significantly decreased (*p* < 0.05 for both). Pharmacological inhibition of p-p65 significantly attenuated KLF7 expression and improved glucose tolerant and insulin sensitive (*p* < 0.05 for both).

**Conclusions:**

Our results indicate that obesity-induced elevated palmitic acid promotes inflammation and glucose metabolism disorders through GPRs/NF-κB/KLF7 signaling pathway.

## Introduction

Obesity is considered as an increasingly common health problem in the world [1]. Excessive free fatty acids (FFAs) links obesity with metabolic complications, such as insulin resistance (IR) and type 2 diabetes mellitus (T2DM) [2, 3]. Palmitic acid (PA) accounts for 27% of total FFAs in plasma [4]. Numerous studies have shown that high level of PA can induce inflammatory response and glucose metabolic disorders [5–11], while the specific mechanism is not fully explicit.

Kruppel-like factors (KLFs) are a group of transcription factors with a zinc finger structure characterized by three C_2_H_2_ zinc finger domains at the carboxy-terminal. Eighteen kinds of KLFs were found so far participating in regulation of proliferation, differentiation, and apoptosis and so on [12, 13]. Among them, KLF7 was found by Matsumoto for the first time in human vascular endothelial cells in 1998 [14]. It was found that KLF7 could promote the expression of inflammatory cytokine Interleukin-6 (IL-6), and inhibit glucose metabolism in human islet cells and HepG2 cells [15, 16]. Meanwhile, our previous studies found KLF7 could transcriptionally activate IL-6 [11]. Furthermore, some researchers suggest KLF7 can regulate specific adipose synthesis and metabolism related genes, such as fatty acid synthase, lipoprotein lipase, fatty acid binding protein 4 [17–19]. Researches above indicate that KLF7 plays vital role in regulation of inflammation, glucose and lipid metabolism. More importantly, our laboratory also found that obesity-induced high concentration of PA could significantly promote KLF7 expression, while the specific mechanism was not elucidated fully [11].

G-protein coupled receptors (GPRs) locate on the cell membrane, among them GPR40, GPR41, GPR43, GPR84 and GPR120 are specifically activated by different FFAs [20–22]. According to the number of carbon atoms in FFAs, they can be divided into short-chain free fatty acids (SCFAs), medium-chain free fatty acids (MCFAs) and long-chain free fatty acids (LCFAs), which bind to different GPRs to activate corresponding signaling pathways: SCFAs can activate GPR41 and GPR43, meanwhile MCFAs and LCFAs work through GPR84, GPR40 and GPR120 [23–27]. Previous studies have shown that PA (C 16:0), belonging to LCFAs, can play physiological roles by activating GPR40 and GPR120. Specifically, it has been reported that PA can combine with GPR40 on the surface of nerve cells to activate the NF-κB signaling pathway, resulting in the release of inflammatory mediators and β-like amyloid to accelerate the occurrence of Alzheimer’s disease [28]. Moreover, PA can promote autophagy by activating GPR40 in neural cell, further reducing insulin sensitivity and leading to the occurrence of T2DM [29]. Han et al. found that FFAs can mediate the c-Raf/MAPK/NF-κB signaling pathway through GPR120 to play a proinflammatory effect in macrophages [30]. The above researches suggest that PA could promote inflammation and glucose metabolism disorders by activating GPR40 and GPR120. Whether PA promotes KLF7 expression depending on GPR40/120 needs to be further demonstrated.

Herein, we first demonstrate PA induces inflammation and glucose metabolism disorders through GPRs/NF-κB/KLF7 signaling pathways in vivo and vitro. More importantly, we found that transcription factor NF-κB significantly activated the expression of KLF7 through luciferase reporter gene experiment and ChIP assay. This study will provide experimental basis and basic theory for clinical treatment of obesity and related metabolic diseases.

## Materials and methods

### Reagents and materials

In total, 40 mM PA solution: PA (Sigma-Aldrich, St. Louis, USA, 0.0614 g) was added to 3 ml NaOH solution (0.1 mol/l), placed in a 75 °C full saponification water bath for 30 min until the PA particles are completely dissolved and the liquid is colorless and transparent. Then the liquid was added to 3 ml BSA (40%, free of fatty acid) solution immediately with sufficient mixing. In total, 100 mM AH7614 solution: AH7614 (TOCRIS, England, 10 mg) was dissolved in 285 μl DMSO. In total, 100 mM GW1100 solution: 5 mg GW1100 (MCE, America, 5 mg) was dissolved in 960 μl DMSO. In total, 10 mM Bay 11-7082 solution: Bay 11-7082 (MCE, America, 5 mg) was dissolved in 2.41 ml DMSO.

### Animal experiment

Animal experiments were approved by the Medical Ethics Committee of the First Affiliated Hospital of Shihezi University School of Medicine (Code: A2019-086-01). Four-weeks-old C57BL/6 male mice were purchased from Slack Jingda Laboratory Animal Company (Hunan) and raised in the animal room of Shihezi University School of Medicine, 3–5 mice/cage were reared. The food and water were changed once a day, the weight and body length were measured weekly. After adaptive feeding for 1 week, mice were divided into normal control diet feeding group (NCD, 10% calories from fat) and high-fat diet feeding group (HFD, 60% calories from fat) randomly. After feeding for 7 weeks, mice in HFD were grouped into four groups: HFD group (DMSO), HFD and intraperitoneal injection of GW1100 (HFD + GW1100 5 mg/kg/day), HFD and intraperitoneal injection of AH7614 (HFD + AH7614, 5 mg/kg/day), HFD and intraperitoneal injection of Bay 11-7082 (HFD + Bay 11-7082, 2.5 mg/kg/day) randomly, the drug injection lasted for 5 weeks.

### Glucose tolerance test and insulin tolerance test

Glucose tolerance test (GTT) and insulin tolerance test (ITT) were conducted at the 12th week. GTT: the feed was removed and water was provided for 12 h, 50% glucose solution (4 μl/g) was injected intraperitoneally, the blood glucose was measured at 0, 0.5, 1, 1.5 and 2 h, respectively. ITT: the feed was removed and the mice were continuously supplied with water for 6 h. The mice were intraperitoneally injected with different doses of insulin (0.5 IU/g), the blood glucose was measured at 0 h, 0.5 h, 1 h, 1.5 h and 2 h, respectively. After GTT and ITT tests, the samples of adipose tissue, liver, and serum were collected for analysis.

### Cell culture

The pre-adipocyte 3T3-L1 was cultured in high glucose Dulbecco’s modified Eagle’s medium (DMEM) with 10% fetal bovine serum (FBS) and 1% penicilinstreptomycin at 37 °C for 48 h under full of atmosphere of 5% carbon dioxide and 95% air. The source of the cell line was identified by STR profiling and tested for mycoplasma contamination. Differentiation was initiated after 2 days with cell growth coverage rate reaching 100%. First, changing culture medium into induce medium I with 0.5 mmol IBMX, 1 μM dexamethasone, and 1 μg/ml insulin for 2 days, then induce medium I was replaced by induce medium II without IBMX and dexamethasone every 48 h for 6–8 days until mature adipocytes was reached about 90%. HepG2 and HEK293T cells were cultured in DMEM with 10% FBS and 1% penicilinstreptomycin at 37 °C under full of atmosphere of 5% carbon dioxide and 95% air.

### Cell treatment

After induction of adipocytes and culturation of HepG2 cells sucessfully in the six-well plate, adding 2 μl DMSO as blank control group, PA group was added with 2 μl DMSO and 10 μl PA storage solution (40 mM) until the final concentration was 200 μM. GW1100 and AH7614 groups were blocked by adding 1, 2, 3 μl GW1100 or AH7614 storage solution to the final concentration of 50, 100, 150 μM, respectively. In total, 1 μl Bay 11-7082 were added to the culture medium until the final concentration was 10 μM to block p-p65.

### Quantitative reverse transcription PCR

Total RNA was isolated from HepG2 cells and 3T3-L1 adipocytes using TRIZOL reagent (Life Technologies, 15596-026, USA). Reverse transcription using Thermo Scientific Revert Aid First Strand cDNA Synthesis Kit (Thermo Scientific, K1622, USA), conditions for cDNA amplification were as follows: 25 °C for 5 min, 42 °C for 60 min, and 70 °C for 15 min.

Real-time PCR was used to measure gene expression using SYBR® Select Master Mix (Applied Biosystems, 4472908, USA), PCR reactions were conducted in 10 μl volumes the detailed PCR reaction were as follow: 94 °C for 5 min, the denaturation temperature was 94 °C for 30 s, the annealing temperature was 53 °C for 30 s, and the extension temperature was 72 °C for 30 s, for a total of 40 cycles. Relative expression levels of related genes were determined by qRT-PCR normalized to GAPDH or β-actin. Primer sequences are available in Table 1.

**Table 1:** Primes sequences used in this study.

genes	5′→3′
Homo-*KLF7*–F	AGCTACAACTTGTCCACGA
Homo-*KLF7*–R	ATTCAAGGCATGTCTGCTG
Homo-*IL-6*-F	AGACAGCCACTCACCTCTTCAG
Homo-*IL-6*-R	TTCTGCCAGTGCCTCTTTGCTG
Homo-*TNF-α*-F	CTCTTCTGCCTGCTGCACTTTG
Homo-*TNF-α*-R	ATGGGCTACAGGCTTGTCACTC
Homo-*MCP-1*-F	AGAATCACCAGCAGCAAGTGTCC
Homo-*MCP-1*-R	TCCTGAACCCACTTCTGCTTGG
Homo-*GLUT4*-F	CCATCCTGATGACTGTGGCTCT
Homo-*GLUT4*-R	GCCACGATGAACCAAGGAATGG
Homo-*RelA*-F	GCTTGTAGGAAAGGACTGC
Homo-*RelA*-R	AGGTTCTGGAAACTGTGGA
Homo-*GAPDH*-F	GGTGGTCTCCTCTGACTTCAA
Homo-*GAPDH*-R	TCTTCCTCTTGTGCTCTTGCT
Mus-*KLF7*–F	GGAAGGATGCGAGTGGCGTTTT
Mus-*KLF7*–R	CGCAAGATGGTCAGACCTGGAG
Mus-*IL-6*-F	TACCACTTCACAAGTCGGAGGC
Mus-*IL-6*-R	CTGCAAGTGCATCATCGTTGTTC
Mus-*TNF-α*-F	GGTGCCTATGTCTCAGCCTCTT
Mus-*TNF-α*-R	GCCATAGAACTGATGAGAGGGAG
Mus-*MCP-1*-F	GCTACAAGAGGATCACCAGCAG
Mus-*MCP-1*-R	GTCTGGACCCATTCCTTCTTGG
Mus-*GLUT4*-F	GGTGTGGTCAATACGGTCTTCAC
Mus-*GLUT4*-R	AGCAGAGCCACGGTCATCAAGA
Mus-*actin*-F	CATTGCTGACAGGATGCAGA
Mus-*actin*-R	CTGATCCACATCTGCTGGAA

### Western blot

The separated proteins were electroblotted onto nitrocellulose filter membrane and blocked for 2 h at room temperature with Tris-buffered saline containing 5% BSA. Nitrocellulose filter membrane were incubated at 4 °C overnight with antibodies, rabbit anti-KLF7 (Abcam; ab197690), rabbit anti-IL-6 (Abcam, ab214429), rabbit anti-GPR120 (Abcam; ab230869), rabbit anti-GPR40 (Thermo Fisher; PA5-75351), rabbit anti-p-IKKβ (Cell Signaling Technology; 2697S), rabbit anti-T-IKKβ (Abcam; ab124957), rabbit anti-p-IκB (Cell Signaling Technology; 2859S), rabbit anti-T-IκB (Cell Signaling Technology; 4812S), rabbit anti-p-p65 (Cell Signaling Technology; 3033S), rabbit anti-T-p65 (Cell Signaling Technology; 8242S), rat anti-GAPDH (ZSGB-BIO; TA-08), rabbit anti-TBP (Cell Signaling Technology; 44059S), rat anti-β-Actin (ZSGB-BIO; TA-10) were used at a dilution of 1:1000. The secondary antibodies (ZSGB-BIO; ZB2301 and ZB2305) were incubated at 25 °C for 2 h at a working ratio of 1:10,000.

### Luciferase reporter gene experiment

Using Eukaryotic Promotor Database software to query the gene sequence of KLF7 promoter region. Jaspar Database predicts the binding site of p65 and KLF7 promoter region. Human KLF7 promoter region core segment luciferase plasmid (2001 bp) and truncated luciferase reporter gene plasmids (1501, 1001, 501 bp) (GenePharma), each luciferase reporter gene plasmid and p65 overexpression plasmid as well as the renilla fluorescent plasmid was co-transfected into HepG2 cells. Testing equipment: full-wavelength scanning multi-functional microplate reader (BioTeK); testing kit: (Promega Dual-Glo® Luciferase Assay System E2920) for sample addition testing in the following order: Add Dual-Glo® Luciferase Assay Reagent to the plate, Incubate at 20–25 °C for 10 min–2 h, Measure firefly luminescence. Add Dual-Glo® Stop & Glo®Reagent to the plate, Incubate at 20–25 °C for 10 min, Measure Renilla luminescence. Calculate ratio of firefly: Renilla luminescence for each well, Normalize the sample well ratio to the ratio from a control (or series of control) wells.

### Chromatin immunoprecipitation assay

In total, 2–4 × 10^6^ cells were crosslinked with 1% formaldehyde for 10 min at room temperature, and then DNA was fragmented into 200–500 bp fragments by Ultrasonic instruments (Sonics & Materials Inc, VCX150, USA). Antibodies specific for p65 (CST, 8242S) or unspecific IgG (CST;3900S) were used for ChIP assay. The DNA was then purified and analyzed by qRT-PCR to detect specific DNA sequences of KLF7 promoter. The sequences of primers are shown in Supplementary Table 1.

### Glucose metabolism assay

HepG2 cells and 3T3-L1 adipocytes were grown in 6-well plates and treated with PA (200 μM) or PA and blocker (100 μM), culture medium was collected for measurement of glucose concentration using the glucose oxidase method (F006-1-1, Nanjing Jiancheng Bioengineering Institute, China).

### Biochemical indicator test

Triglycerides (TG), total cholesterol (TC), high-density lipoprotein cholesterol (HDL-C), and low-density lipoprotein cholesterol (LDL-C) were measured by TC assay kit (A110-1-1, Nanjing Jiancheng Bioengineering Institute, China), TC assay kit (A111-1-1, Nanjing Jiancheng Bioengineering Institute, China), HDL assay kit (A112-1-1, Nanjing Jiancheng Bioengineering Institute, China), and LDL assay kit (A113-1-1, Nanjing Jiancheng Bioengineering Institute, China). PA, IL-6, MCP-1, TNF-α in plasma were detected by mice PA ELISA Kit (A185255, Shanghai Fusheng Bioengineering Institute, China), mice serum IL-6 ELISA Kit (F10830, Shanghai XiTang Bioengineering Institute, China), mice serum MCP-1 ELISA Kit (F11130, Shanghai XiTang Bioengineering Institute, China), mice serum TNF-α ELISA Kit (F11630, Shanghai XiTang Bioengineering Institute, China).

### Statistical analysis

The statistical software SPSS 18.0 was used for data analysis. For the data conform to the normal distribution and similar variance, Student’s *t* test were used when two groups of data were compared. If the comparison between three groups of data, one-way ANOVA-LSD analysis was conducted. *p* < 0.05, the difference is statistically significant. Values are expressed as mean ± SEM (Prism 7; GraphPad Software). In this research, there is no power analysis was performed to determine the sample size. The sample size was based on the previous studies employing in mice. No animals were excluded from the analyses and the blind rule was not used in this study.

## Results

### Effects of PA on GPRs/p-p65/KLF7 levels

GPR120 and GPR40 are G-protein coupled receptors that can bind with LCFAs. A significant increase in GPR40 and GPR120 expression were detected in 3T3-L1 adipocytes (Supplementary Fig. 1a, *p* < 0.05 for both) treatment with 200 μM PA for 48 h and HepG2 cells (Supplementary Fig. 1e, *p* < 0.05 for both) treatment with 200 μM PA for 24 h relative to the control. Meanwhile, after stimulated by 200 μM PA, the expression of phosphorylation p65 (p-p65) and KLF7 were markedly increased in 3T3-L1 adipocytes (Supplementary Fig. 1b, c, *p* < 0.05 for both) and HepG2 cells (Supplementary Fig. 1f, g, *p* < 0.05 for both). Moreover, after treatment with PA, the ability of glucose consumption was impaired in 3T3-L1 adipocytes (Supplementary Fig. 1d, *p* < 0.05) and HepG2 cells (Supplementary Fig. 1h, *p* < 0.05).

### PA regulates NF-κB/KLF7 through GPR40 signaling pathway

**Fig. 1:**
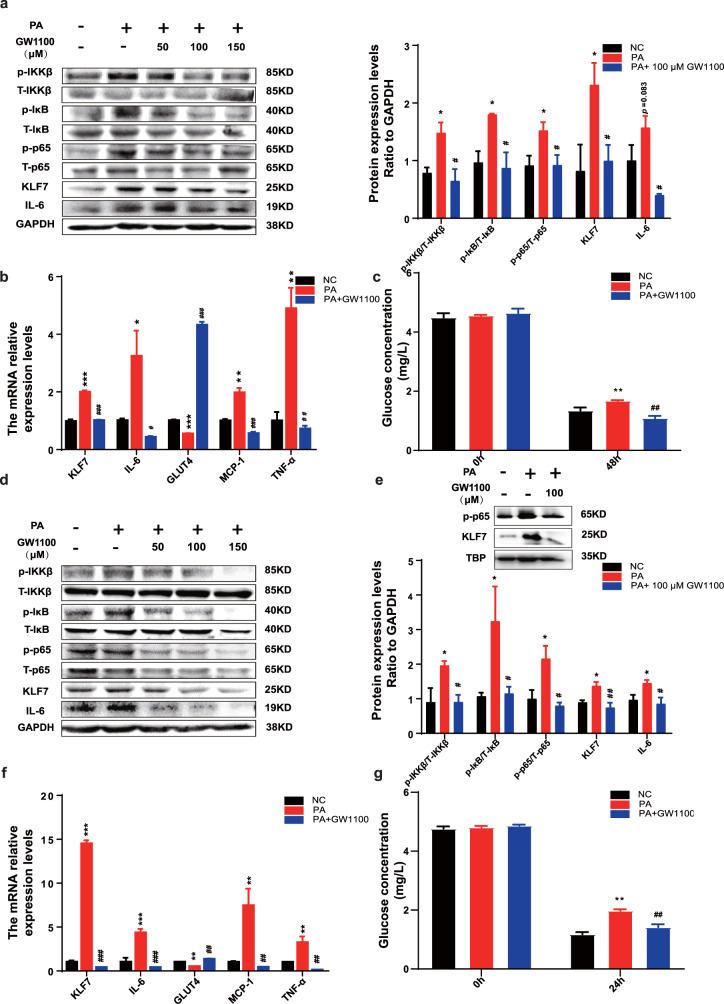
PA regulates KLF7 through GPRs/p-p65 signaling pathway. Add GW1100 (50, 100, 150 μM) to culture medium while stimulated by 200 μM PA, the protein expression levels of p-IKKβ/T-IKKβ, p-IκB/T-IκB, p-p65/T-p65, KLF7 and IL-6 in 3T3-L1 adipocytes (**a**) and HepG2 cells (**d**). The mRNA expression levels of KLF7, IL-6, GLUT4, MCP-1 and TNF-α in 3T3-L1 adipocytes (**b**) and HepG2 cells (**f**). The expression levels of KLF7, p-p65 in HepG2 nuclear extracts (**e**). The glucose consumption ability in 3T3-L1 adipocytes (**c**) and HepG2 cells (**g**). One-way ANOVA-LSD, *NC compared with PA group, ^#^PA compared with GW1100 group. *p < 0.05, **p < 0.01, ***p < 0.001, ^#^*p* < 0.05^, ##^*p* < 0.01, ^###^*p* < 0.001, the difference was statistically significant, data presented as means ± SEM.

The above results demonstrated that PA promoted the expression of GPR40, GPR120, p-p65 and KLF7. To further investigate whether PA regulates p-p65 and KLF7 through GPR40/120 signaling pathway, cells were pre-incubated with GW1100 (an inhibitor of GPR40) and AH7614 (an inhibitor of GPR120) prior to adding PA. Our results showed that GW1100 significantly inhibited PA-stimulated increased phosphorylation levels of IKKβ, IκB, p65, and the protein expression of KLF7, IL-6 whatever in 3T3-L1 adipocytes (Fig. 1a, *p* < 0.05 for both) or HepG2 cells (Fig. 1d, *p* < 0.05 for both). Meanwhile, GW1100 significantly decreased the mRNA expression of KLF7, IL-6, TNF-α as well as MCP-1 in 3T3-L1 adipocytes (Fig. 1b, *p* < 0.05 for both) and HepG2 cells (Fig. 1f, *p* < 0.05 for both). The mRNA expression of GLUT4 was reduced under PA treatment, which was abrogated by GW1100 in 3T3-L1 adipocytes (Fig. 1b, *p* < 0.05 for both) and HepG2 cells (Fig. 1f, *p* < 0.05 for both). Moreover, pharmacological inhibition of GPR40 improve the glucose consumption ability compared with PA group in 3T3-L1 adipocytes (Fig. 1c, *p* < 0.05 for both) and HepG2 cells (Fig. 1g, *p* < 0.05 for both). More importantly, we also found that inhibition of GPR40 could repress the expression levels of p-p65 and KLF7 in HepG2 nucleus (Fig. 1e).

### PA regulates NF-κB/KLF7 through GPR120 signaling pathway

**Fig. 2:**
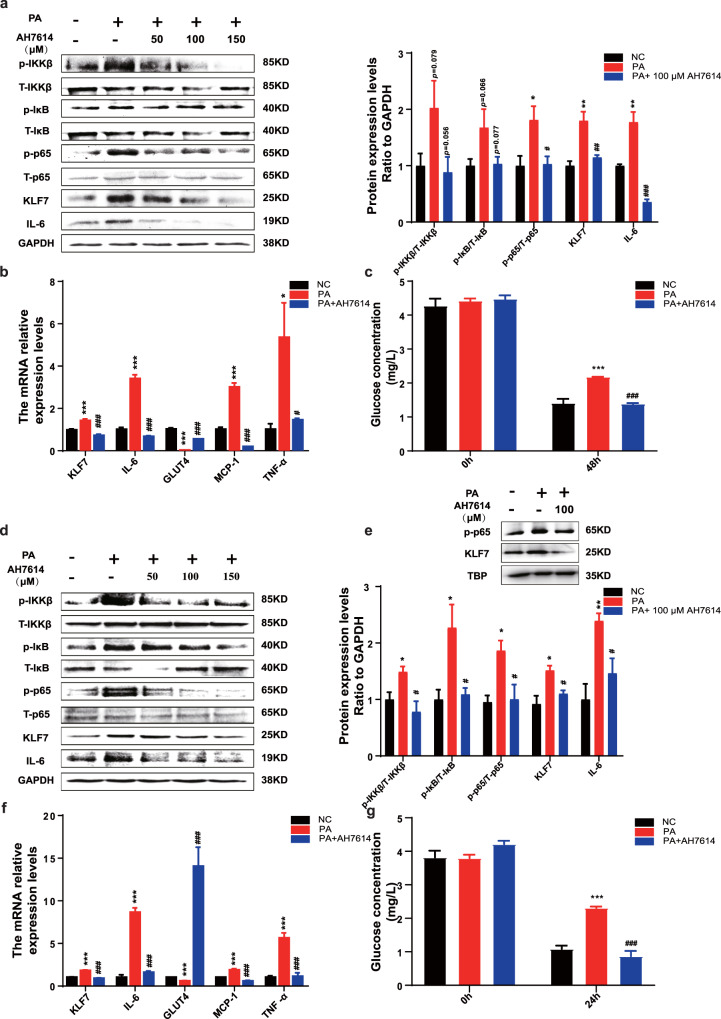
PA regulates KLF7 through GPRs/p-p65 signaling pathway. Add AH7614 (50, 100, 150 μM) to culture medium while stimulated by 200 μM PA, the protein expression levels of p-IKKβ/T-IKKβ, p-IκB/T-IκB, p-p65/T-p65, KLF7 and IL-6 in 3T3-L1 adipocytes (**a**) and HepG2 cells (**d**). The mRNA expression levels of KLF7, IL-6, GLUT4, MCP-1 and TNF-α in 3T3-L1 adipocytes (**b**) and HepG2 cells (**f**). The expression levels of KLF7, p-p65 in HepG2 nuclear extracts (**e**). The glucose consumption ability in 3T3-L1 adipocytes (**c**) and HepG2 cells (**g**). One-way ANOVA-LSD, *NC compared with PA group, ^#^PA compared with AH7614 group. *p < 0.05, **p < 0.01, ***p < 0.001, ^#^*p* < 0.05^, ##^*p* < 0.01, ^###^*p* < 0.001, the difference was statistically significant, data presented as means ± SEM.

The above results demonstrated that PA promoted the expression of KLF7 through GPR40, we next detect whether GPR120 plays role in this progression. Cells were pre-incubated with AH7614 (an inhibitor of GPR120) prior to adding PA. Results showed that AH7614 significantly inhibited phosphorylation levels of IKKβ, IκB, p65, and the protein expression of KLF7, IL-6 whatever in 3T3-L1 adipocytes (Fig. 2a, *p* < 0.05 for both) or HepG2 cells (Fig. 2d, *p* < 0.05 for both). Meanwhile, AH7614 significantly decreased the mRNA expression of KLF7, IL-6, TNF-α as well as MCP-1 in 3T3-L1 adipocytes (Fig. 2b, *p* < 0.05 for both) and HepG2 cells (Fig. 2f, *p* < 0.05 for both). The mRNA expression of GLUT4 was reduced under PA treatment, which was abrogated by AH7614 in 3T3-L1 adipocytes (Fig. 2b) and HepG2 cells (Fig. 2f, *p* < 0.05 for both). Moreover, pharmacological inhibition of and GPR120 improve the glucose consumption ability compared with PA group in 3T3-L1 adipocytes (Fig. 2c, *p* < 0.05 for both) and HepG2 cells (Fig. 2g, *p* < 0.05 for both). Inhibition of GPR120 could also repress the expression levels of p-p65 and KLF7 in HepG2 nucleus (Fig. 2e). In conclusion, these results suggested that PA induced inflammation and glucose metabolism disorders through GPR40 and GPR120.

### PA promotes KLF7 through elevated level of p-p65 and p65 can transcriptionally activate KLF7

**Fig. 3:**
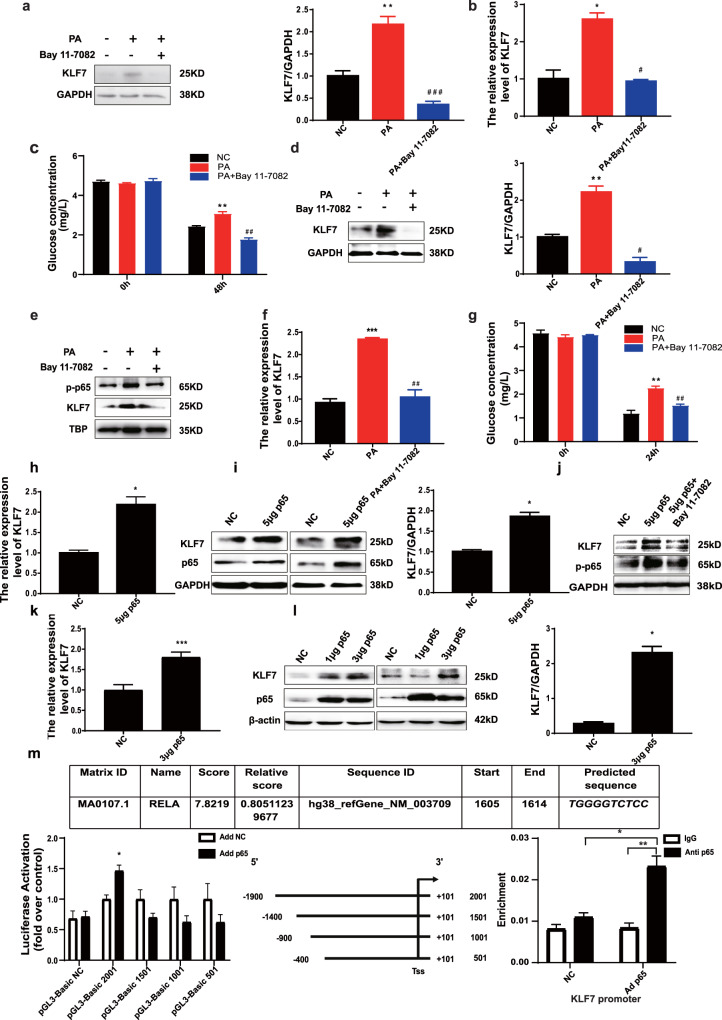
NF-κB has a transcriptional activation effect on KLF7. Add 5 μM Bay 11-7082 to culture medium while stimulated by 200 μM PA to Inhibit the phosphorylation of p65, the protein and mRNA expressions of KLF7 reduced in 3T3-L1 adipocytes (**a**, **b**) and HepG2 cells (**d**, **f**) as well as protein expressions of p-p65 and KLF7 in HepG2 nuclear extracts (**e**). Inhibition of p-p65 improve the glucose consumption ability in 3T3-L1 adipocytes (**c**) and HepG2 cells (**g**). One-way ANOVA-LSD, *NC compared with PA group, ^#^PA compared with Bay 11-7082 group. *p < 0.05, **p < 0.01, ***p < 0.001, ^#^*p* < 0.05, ^##^*p* < 0.01, ^###^*p* < 0.001, the difference was statistically significant, data presented as means ± SEM. p65 overexpression plasmid was transfected to HepG2 and 293T cells, the mRNA and protein expression of p65, KLF7 were increased in HepG2 (**h**, **i**) and 293T cells (**k**, **l**). Blocking the phosphorylation of p-p65 while overexpressed p65, the protein expression levels of p-p65 and KLF7 were detected (**j**). Co-transfecting the human KLF7 promoter region luciferase plasmid and p65 overexpression plasmid into HepG2 cells, detection the luciferase activity value. ChIP assay performed showing enhanced p65 proteins binding to KLF7 gene promoter region (**m**). *t*-test, *p < 0.05, **p < 0.01, ***p < 0.001, the difference was statistically significant, data presented as means ± SEM.

To further explore the potential mechanism of p-p65 in PA-induced KLF7 expression, Bay 11-7082 (an inhibitor of p-p65) was used before incubation with PA. The protein and mRNA expression of KLF7 were significantly lower compared with those of the PA group in 3T3-L1 adipocytes (Fig. 3a, b, *p* < 0.05 for both) and HepG2 cells (Fig. 3d, f, *p* < 0.05 for both). After treatment with Bay 11-7082, the expression of p-p65 and KLF7 in HepG2 nucleus extracts were reduced (Fig. 3e, *p* < 0.05). In addition, pharmacological inhibition of p-p65 improve the glucose consumption ability in 3T3-L1 adipocytes (Fig. 3c, *p* < 0.05) and HepG2 cells (Fig. 3g, *p* < 0.05). The results above indicate that PA promoted the expression of KLF7 by inducing phosphorylation of p65.

After the upregulation of p65 by using the p65 overexpression plasmid, the expression of KLF7 were up-regulated markedly in HepG2 cells (Fig. 3h, i, *p* < 0.05 for both) and HEK293T cells (Fig. 3k, l, *p* < 0.05 for both). The protein expression level of KLF7 was decreased after blocking the phosphorylation of p-p65 while overexpressed p65 (Fig. 3j). Co-transfecting the human KLF7 promoter region luciferase plasmid pGL-3-Basic 2001 and p65 overexpression plasmid into HepG2 cells for 48 h, the luciferase activity value was significantly higher than control group (Fig. 3m, *p* < 0.05), indicating that p65 has a transcriptional activation effect on KLF7. In order to clarify the specific binding site of p65 and KLF7 gene promoter region, we further design 501, 1001 and 1501 bp from the 5’end of the 2001bp sequence of KLF7 promoter region to construct three truncated human KLF7 promoter region luciferase reporters. Co-transfecting KLF7 promoter region luciferase plasmid pGL-3-Basic 1501, 1001, 501 and p65 overexpression plasmid into HepG2 cells, there are no differences between each group (Fig. 3m, *p* < 0.05), indicating that the specific binding site of p65 is located between 1900 and 1400 bp upstream of the transcription start site of KLF7, which is consistent with the results predicted by Jasper Database. Then we performed ChIP assay to detect the binding ability of p65 and KLF7, the result showed that enhanced p65 protein binding to KLF7 gene promoter region (Fig. 3m, *p* < 0.05).

### GPR40/120 antagonist significantly decreased the levels of p-p65 and KLF7 in HFD mice

**Fig. 4:**
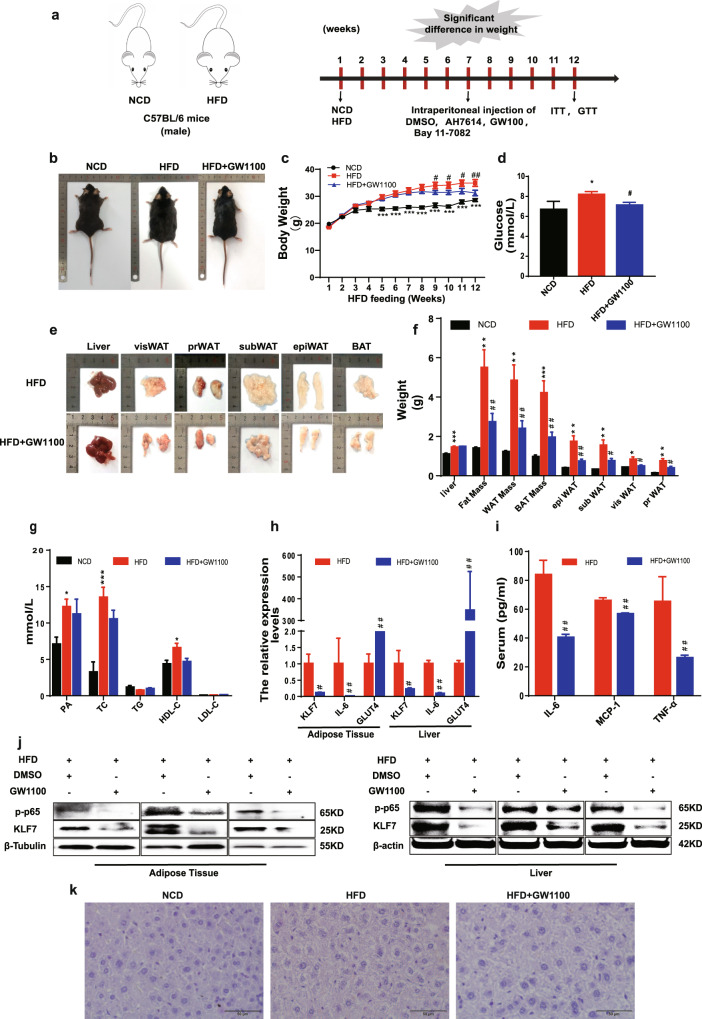
The pharmacological effects of inhibit GPR40. Male C57B/L6 mice were fed with NCD or HFD for 7 weeks, then mice in HFD group were divided into two groups, HFD group and mice intraperitoneally injected with GW1100 (2.5 mg/kg/day) for 5 weeks. The body weight of mice in NCD (*n* = 5), HFD (*n* = 8), and GW1100 (*n* = 8) injection group (**a**–**c**). Weight of liver, epiWAT, subWAT, visWAT, prWAT in NCD, HFD, and GW100 group (**e**, **f**). Blood glucose in NCD, HFD, and GW1100 group (**d**). Plasma levels of palmitic acid, TG, TC, LDL, HDL in NCD, HFD, and GW1100 group (**g**). Protein expression of p-p65 and KLF7 in epiWAT and liver (**j**). mRNA levels of KLF7, IL-6, GLUT4 in epiWAT and liver (**h**). IL-6, MCP-1, TNF-α levels in mice plasma were detected using ELISA assay (**i**), HE staining of liver from mice in NCD, HFD, and GW1100 group (**k**). One-way ANOVA-LSD, *t*-test, *NCD compared with HFD group, ^#^HFD compared with HFD + GW1100 group. *p < 0.05, **p < 0.01, ***p < 0.001, ^#^*p* < 0.05, ^##^*p* < 0.01, ^###^*p* < 0.001, the difference was statistically significant, data presented as means ± SEM.

To further dissect the effects of GPR40 and GPR120 on the levels of p-p65 and KLF7 in vivo, the mice were intraperitoneally injected GW1100 and AH7614. First, C57BL-6J mice were fed either normal chow diet (NCD) or HFD, and the measurement of body weight and serum lipid levels were conducted. Our results revealed that there was a significant enhancement in the body weight (Fig. 4b, c, *p* < 0.05 for both) and serum lipid levels, such as PA, TC, and HDL-C (Fig. 4g, *p* < 0.05) in the HFD mice.

After feeding for 7 weeks, the HFD mice were intraperitoneally injected GW1100 or AH7614 for 5 weeks. In the 12th week, the mice in HFD + GW1100 group represented a significant reduce in body weight compared with HFD group (Fig. 4b, c, *p* < 0.05 for both). Similarly, the weight of epididymal white adipose tissue (epiWAT), perirenal WAT, visceral WAT, as well as subcutaneous WAT were substantially reduced (Fig. 4e, f, *p* < 0.05 for both). However, there were no significant differences in PA, TC, TG, HDL-C, and LDL-C between HFD and HFD + GW1100 group (Fig. 4g, *p* < 0.05). Plasma glucose content was decreased (Fig. 4d, *p* < 0.05), and serum IL-6, MCP-1, TNF-α were modesty decreased in HFD + GW1100 group (Fig. 4i, *p* < 0.05). Western blot analysis demonstrated that compared with HFD mice, the protein expression levels of KLF7, p-p65 were inhibited (Fig. 4j), mRNA levels of KLF7, IL-6 were decreased, and the mRNA expression of GLUT4 was higher in epiWAT and liver of HFD + GW1100 group (Fig. 4h, *p* < 0.05). HE staining of liver showed no difference between HFD and HFD + GW1100 (Fig. 4k).

**Fig. 5:**
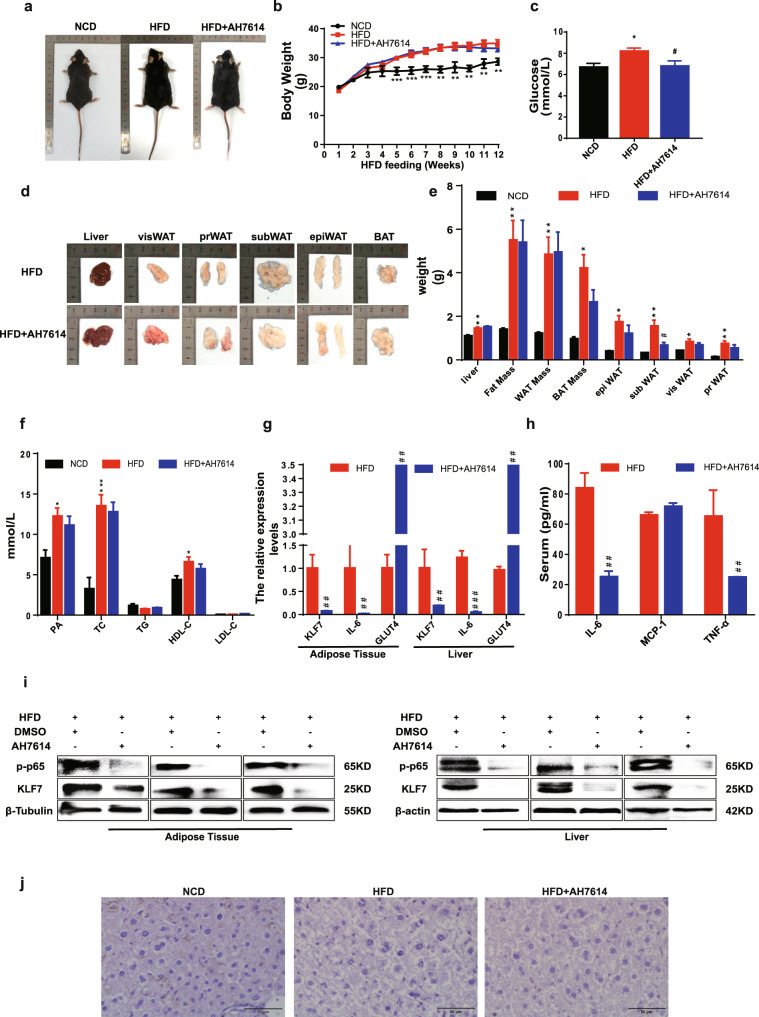
The pharmacological effects of inhibit GPR120. Male C57B/L6 mice were fed with NCD or HFD for 7 weeks, then mice in HFD group were divided into two groups, HFD group and mice intraperitoneally injected with AH7614 (2.5 mg/kg/day) for 5 weeks. The body weight of mice in NCD (*n* = 5), HFD (*n* = 8), and AH7614 group (*n* = 8) (**a**, **b**). Weight of liver, epiWAT, subWAT, visWAT, prWAT in NCD, HFD, and AH7614 group (**d**, **e**). Blood glucose of mice in NCD, HFD, and AH7614 group (**c**). Plasma levels of palmitic acid, TG, TC, LDL, HDL of mice in NCD, HFD, and AH7614 group (**f**) (*n* = 5–8). Protein expression of p-p65 and KLF7 in epiWAT and liver (**i**). mRNA levels of KLF7, IL-6, GLUT4 in epiWAT and liver (**g**). IL-6, MCP-1, TNF-α levels in mice plasma were detected using ELISA (**h**) HE staining of liver from mice in NCD, HFD, and AH7614 group (**j**). One-way ANOVA-LSD, *t*-test, *NCD compared with HFD group, ^#^HFD compared with HFD + AH7614 group. *p < 0.05, **p < 0.01, ***p < 0.001, ^#^*p* < 0.05, ^##^*p* < 0.01, ^###^*p* < 0.001, the difference was statistically significant, data presented as means ± SEM.

Compared HFD group with HFD + AH7614 group, there was no obvious difference in body weight (Fig. 5a, b, *p* < 0.05 for both), the weight of adipose tissues as well as PA, TC, TG, HDL-C, and LDL-C (Fig. 5d–f, *p* < 0.05 for both). Glucose content and serum IL-6, MCP-1, TNF-α were decreased (Fig. 5c, h, *p* < 0.05 for both), and the protein expression levels of KLF7, p-p65 were reduced in epiWAT and liver (Fig. 5i). mRNA levels of KLF7, IL-6 in epiWAT and liver in HFD + AH7614 group were lower than HFD group, the mRNA expression of GLUT4 was higher than HFD mice (Fig. 5g, *p* < 0.05). HE staining of liver showed no difference between HFD and HFD + AH7614 group (Fig. 5j). The results above demonstrated that GPR40/120 antagonist significantly decreased the levels of p-p65 and KLF7 in HFD mice.

### Pharmacological inhibition of p-p65 attenuated KLF7 expression in HFD mice

**Fig. 6:**
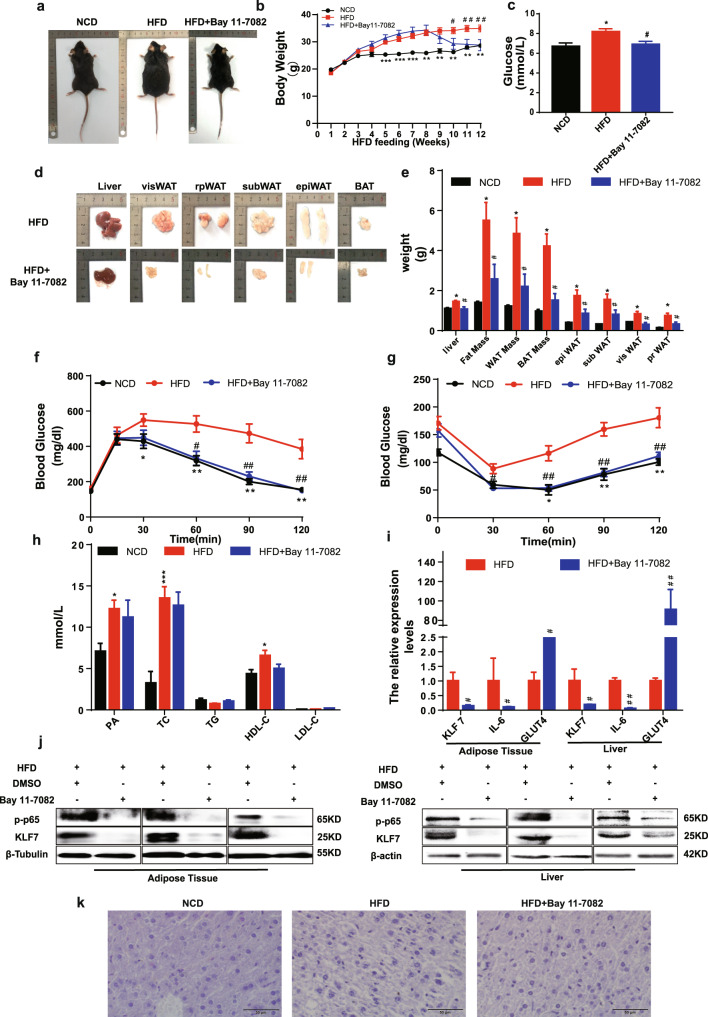
The pharmacological effects of inhibit NF-κB. Male C57B/L6 mice were fed with NCD or HFD for 7 weeks, then mice in HFD group were divided into two groups, HFD group and mice intraperitoneally injected with Bay 11-7082 (2.5 mg/kg/day) for 5 weeks. Dynamic changes in body of NCD, HFD, and Bay 11-7082 group (**a**, **b**). Weight of liver, epiWAT, subWAT, visWAT, prWAT in NCD (*n* = 5), HFD (*n* = 8), and Bay 11-7082 group (*n* = 6) (**d**, **e**). Blood glucose, GTT and ITT in NCD, HFD, and Bay 11-7082 group (**c**, **f**, **g**). Plasma levels of palmitic acid, TG, TC, LDL, HDL (**h**). Western blot of p-p65 and KLF7 in the murine epiWAT and liver (**j**). mRNA levels of KLF7, IL-6, GLUT4 in epiWAT and liver (**i**). HE staining of liver from mice (**k**). One-way ANOVA-LSD, *t*-test, *NCD compared with HFD group, ^#^HFD compared with HFD + Bay 11-7082 group. *p < 0.05, **p < 0.01, ***p < 0.001, ^#^*p* < 0.05, ^##^*p* < 0.01, ^###^*p* < 0.001, the difference was statistically significant, data presented as means ± SEM.

We examined in more detail the potential effects of p-p65 on KLF7 in vivo. The body weight of mice in the HFD + Bay 11-7082 group significantly decreased in contrast to the HFD group (Fig. 6a, b, d, e, *p* < 0.05 for both). In addition, we measured serum glucose tolerance and insulin sensitivity in mice by GTT and ITT. Following the injection of glucose or insulin, the HFD + Bay 11-7082 group mice were much more glucose tolerant and insulin sensitive than HFD littermates (Fig. 6c, f, g, *p* < 0.05 for both), there were no significant differences in TC, TG, HDL, LDL between HFD and HFD + Bay 11-7082 group (Fig. 6h, *p* < 0.05). epiWAT and liver from HFD + Bay 11-7082 group mice expressed a much lower level of KLF7, IL-6, p-p65 and higher GLUT4 compared with HFD group mice (Fig. 6i, j, *p* < 0.05 for both). HE staining of liver showed no difference between HFD and HFD + Bay 11-7082 group (Fig. 6k). These findings suggested that pharmacological inhibition of p-p65 significantly attenuated KLF7 expression in HFD mice.

## Discussion

Numerous studies have shown that elevated serum FFAs level is a key factor in obesity-induced inflammation, IR, and T2DM. According to the number of carbon atoms and the degree of saturation, FFAs can be classified into various types, and play different roles in many diseases [31]. As a LCFA, PA accounts for 27% of the total FFAs in human, which can be provided through diet or synthesized de novo in the body [32]. It has been reported that PA could induce inflammatory response and glucose metabolic disorders [33, 34]. Consistent with previous studies, our results also found that the content of PA in the serum and the expression of inflammatory related factors were significantly increased in HFD mice, while glucose tolerance and insulin sensitivity were significantly impaired. In vitro, we also found that high concentration of PA could significantly promote inflammatory respond and impair the ability of glucose consumption in HepG2 cells and 3T3-L1 adipocytes.

Our previous results have shown that PA can regulate inflammatory response by up-regulating the expression of KLF7 [11]. Moreover, it has been reported that KLF7 plays a vital role in regulation of inflammation, glucose and lipid metabolism [15, 17, 18]. This study was also confirmed that PA could significantly promoted the expression of KLF7 in HepG2 cells and 3T3-L1 adipocytes. Therefore, our results and previous studies demonstrated that PA could induce inflammatory response and glucose metabolic disorders by promoting KLF7, while the specific molecular mechanism needs to be further explored.

FFAs can bind with GPRs to perform various function, including GPR40, GPR120, GPR41, GPR43 and GPR84, among which GPR40 and GPR120 can be activated by PA [35–38]. Studies have shown that the role of GPR40/120 activation is controversial in the occurrence of obesity, inflammation and T2DM. Usui et al. found that after stimulated by FFAs, GPR40 and GPR120 on the surface of pancreatic β-cells can activate ERK1/2 and PI3K/Akt signaling pathways to inhibit inflammatory response and improve IR as well as glucose homeostasis [36, 39, 40]. But with the in-depth study, more and more results indicate that GPR40 and GPR120 do not play a beneficial role fully. Up-regulating the expression of GPR40 in mice fed with HFD impaired the insulin secretion of pancreatic β-cells, and knocking out GPR40 can significantly improve the IR of mice [41]. Hernández et al. found that PA can promote the phosphorylation of p65 by activating GPR40, inducing autophagy and reducing insulin sensitivity in hypothalamic neuronal cells [42]. In adipocytes from morbidly obese subjects, TNF-α and IL-6 expression were significantly increased under PA stimulation, while the effects were reversed by silencing GPR120 [43]. In this study, after pharmacological inhibition of GPR40 and GPR120 respectively, it was found that the expression levels of KLF7, IL-6, MCP-1 and TNF-α were significantly reduced, the expression of GLUT4 was significantly increased, and improved the impairment of cell glucose consumption caused by PA in 3T3-L1 adipocytes and HepG2 cells. After intraperitoneal injection of GPR40 and GPR120 blockers to HFD mice, the expression of KLF7 in adipose and liver tissues and the inflammatory factors in serum, such as IL-6, TNF-α and MCP-1, were all significantly elevated. While, the expression of GLUT4 was significantly reduced and the increasing blood glucose caused by HFD was improved. The above results suggest that PA can up-regulate the expression of KLF7 by activating GPR40 and GPR120 in adipose and liver tissues, inducing inflammation and disordered glucose metabolism.

As transmembrane receptors, GPRs consists of seven transmembrane structures, which can be divided into intracellular and extracellular structures [44]. The intracellular part contains the G-protein binding region, including G-protein α, β and γ, which binds to GDP in resting state [45]. In general, the downstream signaling pathway activated by GPRs depends on the type of α subunit, and the selection of α subunit by GPRs mainly depends on the type of ligand to which GPRs binds, including Gαs, Gαi/o, Gαq/11 and Gα12/13 [46–48]. When PA activates GPR40 and GPR120, which downstream α subunits can play a critical physiological function needs to be further studied.

NF-κB signaling pathway can participate in the occurrence of inflammation and then closely related to the development of T2DM. Studies have shown that after activated by FFAs, GPR40/120 exert proinflammatory effect mainly through NF-κB signaling pathway. For instance, EPA and DHA can activate the c-Raf/MAPK/NF-κB signaling pathway through GPR120 on the surface of macrophages to mediate the release of inflammatory factors [30, 47]. PA can activate GPR40 to promote the phosphorylation of p65, leading to autophagy of neuronal cells and reduce insulin sensitivity [42]. In addition, PA can activate Akt/NF-κB signaling pathway through GPR40 to promote the production of β-like amyloid, accelerating the Alzheimer’s occurrence and development [28]. In this study, after pharmacological inhibition of GPR40/120, the expressions of p-IKKβ, p-IκB and p-p65 protein in 3T3-L1 adipocytes and HepG2 cells were significantly inhibited. After intraperitoneal injection of GPR40 and GPR120 blockers in diet-induced obese mice, the expressions of p-p65 in adipose tissue were significantly reduced. In accordance with previous literatures, these above results suggests that PA can active NF-κB signal pathway by GPR40/120.

More importantly, after intraperitoneal injection of Bay 11-7082, this study found that the expression levels of KLF7 and IL-6 in adipose and liver tissue were significantly reduced and the disorder of glucose metabolism was significantly improved. In 3T3-L1 adipocytes and HepG2 cells, we found that PA promoted KLF7 by increasing the level of p-p65. Luciferase reporter gene results showed that p65 can transcriptionally activate KLF7 expression, and the specific binding site of p65 is located between 1900 and 1400 bp upstream of the transcription start site of KLF7. The above results intensely indicate that p65 can transcriptionally activate KLF7. It is common knowledge that NF-κB is a key classical transcription factor in regulating inflammation, which promotes the expression of most inflammatory factors, such as IL-6, TNF-α, and IL-1β [49–51]. Moreover, our previous study indicated that KLF7 could transcriptionally activate IL-6 [11]. This study found that KLF7 is also regulated by NF-κB for the first time. We can reasonably deduce that KLF7 may strengthen the proinflammatory effect of NF-κB. Interestingly, we have already found that overexpression of KLF7 in 3T3-L1 adipocytes can also significantly promote phosphorylation of p65 [11]. Thus, there may be a mutual regulation relationship between p65 and KLF7, which requires in-depth exploration in the future.

**Fig. 7:**
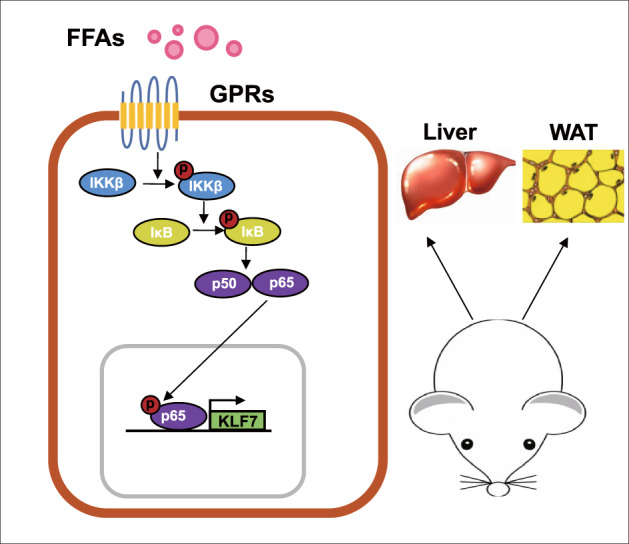
Pattern diagram. Obesity-induced elevated palmitic acid can activate GPRs/NF-κB signaling pathway in liver and adipose tissue, up-regulate the expression of KLF7, and ultimately lead to glucose metabolism disorders.

In summary, this study shows that increasing PA content after obesity induces inflammation and glucose metabolism disorders through GPRs/NF-κB/KLF7 signaling pathways (Fig. 7). This study will provide experimental data and theoretical basis for revealing the high level of FFAs leading to inflammation, IR and other related metabolic diseases.

## Supplementary information

Supplementary figure 1

supplementary table1

## Data Availability

The data used in this study are available from the corresponding author on reasonable request.
