# Supplementary material for: Obesity-induced elevated palmitic acid promotes inflammation and glucose metabolism disorders through GPRs/NF-κB/KLF7 pathway
Source: Nutr Diabetes. 2022 Apr 20;12:23. doi: 10.1038/s41387-022-00202-6 (PMC9021212; doi:10.1038/s41387-022-00202-6)
Supplement: Supplementary file 1 — Supplementary figure 1 [file 41387_2022_202_MOESM1_ESM.docx]

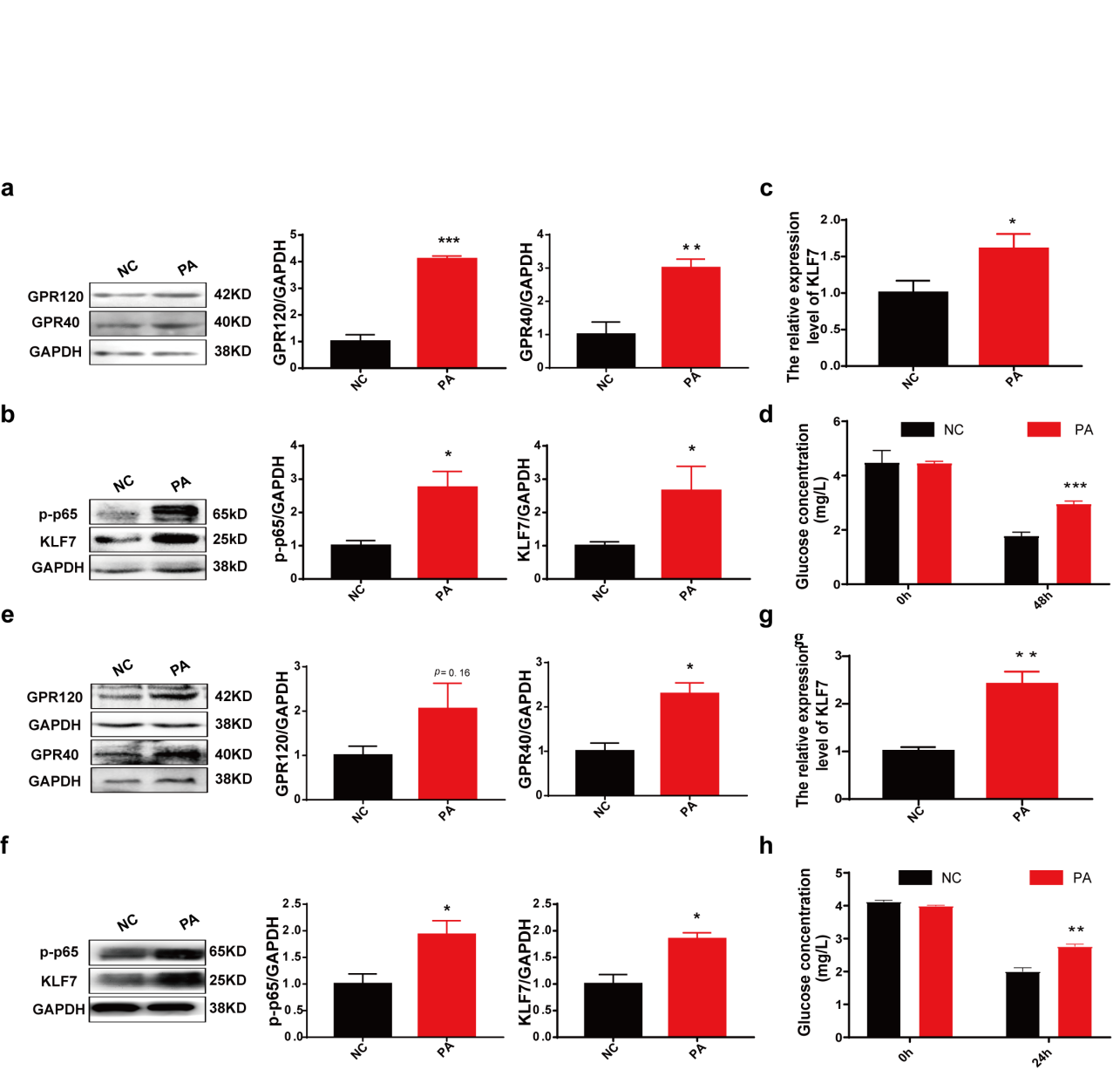
**Supplementary Fig. 1.** **The effect of PA on the regulation of GPRs/p-p65/KLF7 in 3T3-L1 adipocytes and HepG2 cells.** The expression of KLF7, p-p65, GPR40 and GPR120 in 3T3-L1 adipocyte (a-c) and HepG2 cell (e-g) stimulated by 200 μM PA. The glucose consumption ability of 3T3-L1 adipocyte (d) and HepG2 cell (h) was detected. t-test, **P* < 0.05, ***P* < 0.01, ****P* < 0.001, the difference was statistically significant, data presented as means ± SEM.
