# Supplementary material for: Obesity-induced elevated palmitic acid promotes inflammation and glucose metabolism disorders through GPRs/NF-κB/KLF7 pathway
Source: Nutr Diabetes. 2022 Apr 20;12:23. doi: 10.1038/s41387-022-00202-6 (PMC9021212; doi:10.1038/s41387-022-00202-6)
Supplement: Supplementary file 2 — supplementary table1 [file 41387_2022_202_MOESM2_ESM.docx]

**Table1**

Primes sequences used in this study

| genes | 5’→3’ |
| --- | --- |
| Homo-*KLF7*–F | AGCTACAACTTGTCCACGA |
| Homo-*KLF7*–R | ATTCAAGGCATGTCTGCTG |
| Homo-*IL-6*-F | AGACAGCCACTCACCTCTTCAG |
| Homo-*IL-6*-R | TTCTGCCAGTGCCTCTTTGCTG |
| Homo-*TNF-α*-F | CTCTTCTGCCTGCTGCACTTTG |
| Homo-*TNF-α*-R | ATGGGCTACAGGCTTGTCACTC |
| Homo-*MCP-1*-F | AGAATCACCAGCAGCAAGTGTCC |
| Homo-*MCP-1*-R | TCCTGAACCCACTTCTGCTTGG |
| Homo-*GLUT4*-F | CCATCCTGATGACTGTGGCTCT |
| Homo-*GLUT4*-R | GCCACGATGAACCAAGGAATGG |
| Homo-*RelA*-F | GCTTGTAGGAAAGGACTGC |
| Homo-*RelA*-R | AGGTTCTGGAAACTGTGGA |
| Homo-*GAPDH*-F | GGTGGTCTCCTCTGACTTCAA |
| Homo-*GAPDH*-R | TCTTCCTCTTGTGCTCTTGCT |
| Mus-*KLF7*–F | GGAAGGATGCGAGTGGCGTTTT |
| Mus-*KLF7*–R | CGCAAGATGGTCAGACCTGGAG |
| Mus-*IL-6*-F | TACCACTTCACAAGTCGGAGGC |
| Mus-*IL-6*-R | CTGCAAGTGCATCATCGTTGTTC |
| Mus-*TNF-α*-F | GGTGCCTATGTCTCAGCCTCTT |
| Mus-*TNF-α*-R | GCCATAGAACTGATGAGAGGGAG |
| Mus-*MCP-1*-F | GCTACAAGAGGATCACCAGCAG |
| Mus-*MCP-1*-R | GTCTGGACCCATTCCTTCTTGG |
| Mus-*GLUT4*-F | GGTGTGGTCAATACGGTCTTCAC |
| Mus-*GLUT4*-R | AGCAGAGCCACGGTCATCAAGA |
| Mus-*actin*-F | CATTGCTGACAGGATGCAGA |
| Mus-*actin*-R | CTGATCCACATCTGCTGGAA |
| Proximal promoter region F of *KLF7*: | ATTTCCAAGCGTGGGTATCT |
| Proximal promoter region R of *KLF7*: | GTATGCGCATAAACACTTTA |
